# Barriers to access and utilization of healthcare services for minority-language speakers with neurodevelopmental disorders: A scoping review

**DOI:** 10.3389/fpsyt.2022.915999

**Published:** 2022-08-25

**Authors:** Myriam L. H. Beauchamp, Kaela Amorim, Samantha N. Wunderlich, Jonathan Lai, Julie Scorah, Mayada Elsabbagh

**Affiliations:** ^1^Montreal Neurological Institute, McGill University, Montreal, QC, Canada; ^2^Autism Alliance of Canada and Institute of Health Policy, Management and Evaluation, Dalla Lana School of Public Health, University of Toronto, Toronto, ON, Canada

**Keywords:** healthcare access, bilingual, neurodevelopmental disorders, minority-language speakers, multilingual, healthcare disparities

## Abstract

**Introduction:**

Minority-language speakers in the general population face barriers to accessing healthcare services. This scoping review aims to examine the barriers to healthcare access for minority-language speakers who have a neurodevelopmental disorder. Our goal is to inform healthcare practitioners and policy makers thus improving healthcare services for this population.

**Inclusion criteria:**

Information was collected from studies whose participants include individuals with a neurodevelopmental disorder (NDD) who are minority-language speakers, their family members, and healthcare professionals who work with them. We examined access to healthcare services across both medical and para-medical services.

**Method:**

Searches were completed using several databases. We included all types of experimental, quasi-experimental, observational and descriptive studies, as well as studies using qualitative methodologies. Evidence selection and data extraction was completed by two independent reviewers and compared. Data extraction focused on the barriers to accessing and to utilizing healthcare for minority-language speakers with NDDs. The search process and ensuing results were fully reported using a diagram from the *Preferred Reporting Items for Systematic Reviews and Meta-analyses extension for scoping review*.

**Results:**

Following the database search, a total of 28 articles met our final selection criteria and two articles were hand-picked based on our knowledge of the literature, for a total of 30 articles. These studies revealed that minority-language speakers with NDDs and their families experience several barriers to accessing and utilizing healthcare services. These barriers, identified at the Systems, Provider and Family Experience levels, have important consequences on children's outcomes and families' well-being.

**Discussion:**

While our review outlined several barriers to access and utilization of healthcare services for minority-language speakers with NDDs and their families, our findings give rise to concrete solutions. These solutions have the potential to mitigate the identified barriers, including development and implementation of policies and guidelines that support minority-language speakers, practitioner training, availability of referral pathways to appropriate services, access to tools and other resources such as interpretation services, and partnership with caregivers. Further research needs to shift from describing barriers to examining the efficacy of the proposed solutions in mitigating and eliminating identified barriers, and ensuring equity in healthcare for minority-language speakers with NDDs.

## Introduction

Worldwide, there are more bilingual speakers than monolingual speakers (1) and many of these individuals live in environments where their “mother tongue” is a minority language [i.e., a language not spoken by most people in a given environment; (2)]. These minority-language speakers often face barriers to access or utilization of healthcare services (3). This is true regardless of whether they are speakers of an official minority language or a non-official minority language (4, 5). A key barrier encountered by minority language speakers is discordance between them and their healthcare practitioner's preferred or known language, which in turn can lead to communication breakdowns that can have important consequences for patients' health and well-being (6). Other barriers include the use of ad hoc interpreters (7), a reduced number of available services (8, 9), and barriers linked to negative attitudes held by healthcare workers regarding minority-language speakers (7, 10). Additionally, in the context of pediatric healthcare, parents of children from minority-language families also face barriers. Specifically, parents with limited proficiency in the majority language are reported to have more difficulty accessing healthcare services for their child, face challenges in communicating with healthcare practitioners, and are also more likely to misunderstand their child's diagnosis and treatment plan (11).

Barriers facing minority language speakers are amplified in the presence of neurodevelopmental disorders (NDD). Such conditions appear at birth or early in life and have an impact on development across the lifespan (12). This class of conditions includes autism spectrum disorder, intellectual disability, communication disorders, learning disabilities and motor disorders (12). A recent report indicates that 17% of children have been diagnosed with an NDD (13). For these children and their families, barriers to equal access and utilization of healthcare services have been linked to social determinants such as ethnicity, race and immigration status. These children and their families experience important disparities in accessing diagnostic and intervention services (14). Ethnicity has been linked to delays in accessing diagnostic services (15), which then delays children's ability to access intervention services. It has also been linked to reduced number of services, higher levels of unmet needs, particularly with regards to intensive intervention services, respite care, and psychological services (15). Similarly, children from immigrant families tend to access autism services at a later age than non-immigrant children (16) and their parents often report feelings of isolation and loneliness (17). In addition, ethnically diverse families report having access to fewer sources of information, a lack of resource material, and fewer social supports (15, 18). Professionals also receive little or no training in cultural sensitivity, even when they frequently work with culturally diverse populations (18). Moreover, the lack of access to interpreters is also a barrier to offering healthcare services that are culturally sensitive and appropriate (18). Finally, limited proficiency in the majority language makes it much more challenging for immigrant families to navigate the service delivery systems (14).

While studies clearly show that social determinants such as immigration status, ethnicity and race are linked to disparities in services for individuals with NDDs and their families, relatively few studies have focused specifically on the link between being a minority-language speaker with an NDD and barriers to healthcare access and utilization. Indeed, much of the research on minority-language speakers with NDD has instead focused on whether children with NDDs should learn more than one language. Traditionally, the belief has been that for children who already have challenges acquiring one language, the presence of a second language would likely increase language delays or that it would somehow confuse the child (19). Such erroneous beliefs have led to parents from minority-language backgrounds to avoid the use of their minority language with their child, which can have negative consequences on child-parent communication and can limit language-learning opportunities [(20), see (19) for discussion]. Moreover, many healthcare practitioners appear to share these beliefs and recommend against bilingualism for these children (20). In contrast, research over the past 20 years indicates that children with various NDDs can become bilinguals and that they can develop language abilities similar to those of their monolingual peers with similar developmental profiles, in at least one of their languages. This is true for children on the autism spectrum (21, 22), with a developmental language disorder [DLD, formally Specific Language Impairment; (23)], Down's Syndrome (24), and other NDDs. However, bilinguals do not always have monolingual-like abilities in both of their languages. Indeed, bilinguals' abilities in each of their languages are seldom equal (1) and are influenced by several factors, most notably the amount of exposure that children receive to each of their languages (25–28). Therefore, in children who are exposed to more than one language, language abilities in one of their languages that differ from the monolingual norm could indicate a language disorder, but could equally indicate a lack of exposure to that language.

The aim of the current scoping review is to identify the barriers to accessing and utilizing healthcare services for individuals with NDDs who are minority-language speakers. Examining barriers specific to minority language speakers is important for several reasons. First, this subgroup is not always captured in the social determinants of race, ethnicity, or immigration status. Additionally, race, ethnicity, and immigration status can be linked to being a language minority but are not necessarily so. Thus, examining the influence of being a minority-language speaker as a stand-alone social determinant will shed light on barriers specific to this minority group.

## Method

A preliminary search of Embase, Google Scholar and PsychINFO was completed on December 3rd, 2021, and there were no published scoping or systematic reviews on this subject. A search of the Open Science Framework (OSF) on December 6th, 2021, also revealed no registered ongoing studies or publications in preprint examining our research aim. Our research protocol was published on the OSF platform on March 1st, 2022, prior to data extraction commencing.

### Types of sources

For this review, we considered all types of experimental and quasi-experimental study design types. We also included observational studies and descriptive studies. In addition, qualitative studies including phenomenological interviews were included. Reviews were excluded, as were conference posters and conference talks, and unpublished (gray) literature. Moreover, for the purposes of this review, no language or date restriction criteria was imposed.

### Eligibility criteria

To be considered for this review, studies were required to meet the following criteria:

A) Population: Studies were considered if they include individuals with NDDs who were minority-language speakers of any age, or caregivers of people with NDDs who are minority-language speakers. Additionally, study participants were required to be defined as minority-language speakers, bilinguals, multilinguals or as having limited English proficiency. A full list of the population criteria can be found in Table 1.
B) Condition: Following the DSM-5 (12), we defined NDDs as follows: Autism Spectrum Disorder [ASD; we also included in this category Pervasive Developmental Disorder-Not Otherwise Specified (PDD-NOS) and Asperger's since these terms would have been used in studies prior to changes in the DSM-5 published in 2013], Attention Deficit Disorder (with and without hyperactivity; ADD and ADHD), Intellectual Disability, Communication Disorder [including a Developmental Language Disorder, Speech Sounds Disorders, Social Communication (Pragmatic) Disorder], and Learning Disorders. A full list of the conditions can be found in Table 1. Additionally, we defined minority-language speakers as individuals who speak a language other than the majority language (i.e., the language spoken by most individuals in a given environment.) using the following terms: bilingual, multilingual, minority language, and limited English proficiency. We included the terms “bilingual” and “multilingual” since we consider that, in most cases minority-language speakers will be (or need to become) minimally bilingual.
C) Context: Any context in which formal healthcare services are delivered by doctors, nurses, speech-language pathologists (SLPs), psychologists, social workers, occupational therapists, physiotherapists, or early interventionists, including within medical and academic contexts, and within the home. In addition, we defined healthcare access to services as the ability to obtain services from any medical or paramedical professional including doctors, nurses, speech-language pathologists, psychologists, social workers, occupational therapists, physiotherapists. We also included the terms *therapists, intervention, interventionist, healthcare, health services* and *healthcare services*. Given that some healthcare services are offered within the school system, we included access to such services in schools by combining the above terms with the term *school*. A full list of the contexts can be found in Table 1.

**Table 1:** Inclusion and exclusion criteria for studies.

**Category**	**Inclusion**	**Exclusion**
Population	• Bilingual, minority-language speakers, minoritized-language speakers AND • Disorders (Neurodevelopmental, ASD, Asperger's, PDD-NOS, ADD, ADHD, Down's Syndrome, Global developmental delay, Intellectual Disability, Language Disorder, Language Impairment, Communication Disorder (Developmental Language Disorder, Speech Sounds Disorders, Social Communication (Pragmatic) Disorder), and Learning Disorders)	• Other NDDs beyond our inclusion criteria
Context	• Clinical services in healthcare or educational settings, e.g., medical doctors, SLPs, SLTs, psychologists, social workers, doctors, nurses, occupational therapists, physiotherapists, and administrators • Early educators (ABA therapists, psycho-educator etc.) • Parents and caregivers of individuals with NDDs • Individuals with NDDs	• Other school workers (E.g., teachers and educational assistants)
Types of evidence	• Original research	• Reviews • Conference abstracts or posters • Government documents • Dissertations • Other “gray” literature
Concept	• Actual or perceived barriers to healthcare or educational services, e.g., beliefs about second language access or ability in children with NDDs • Knowledge of bilingualism in individuals with NDDs • Access or utilization of healthcare for minoritized language speakers with NDD	

### Search strategy

Based on prior knowledge of the literature, and following a MeSH term search, we developed an initial list of terms to designate NDDs, minority-language speakers, as well as a list of contexts (such as healthcare) where barriers to accessing and utilizing healthcare services could occur. Next, we completed an initial search using PubMed to identify articles examining healthcare service access for minority-language speakers. We validated and adjusted our initial list by comparing our search terms to (a) the keywords from abstracts and titles, (b) the keywords from the indexing list and (c) the MeSH terms linked to 80 existing studies that dealt with our three themes (NDDs, minority-language speakers, healthcare access). Our final list of search terms can be found in Table 2. Searches were completed using the following databases: PubMed, Embase, PsychINFO, Scopus, and CINAHL.

**Table 2:** Example of SCOPUS search terms.

**Search** **line**	**Search string**	**Concept**
1	TITLE-ABS-KEY(“neurodevelopmental disorders” OR “neurod#v*” OR “NDD” OR “autism” OR “ASD” OR “Aspergers” OR “PDD-NOS” OR “pervasive developmental disorder-not otherwise specified” OR “ADHD” OR “attention deficit hyperactivity disorder” OR “GDD” OR “global developmental delay” OR “intellectual disabilit*” OR “intellectual disability” OR “LD” OR “language disorder*” OR “language impairment” OR “communication disorder*” OR “learning disorder”)	Neurodevelopmental Disorder
2	TITLE-ABS-KEY(“bilingual*” OR “minority language*” OR “limited English” OR “multilingual*” OR “minoritized language”)	Language
3	TITLE-ABS-KEY(“healthcare” OR “school*” OR “doctor*” OR “Social worker*” OR “Medical*” OR “Clinician*” OR “SLP” OR “Speech language patholo*” OR “Nurse*” OR “psycholog*” OR “Therap*” OR “Treat*” OR “Health service*” OR “Health care” OR “Intervent*” OR “Practitioner*” OR “Professional*” OR “parent*” OR “caregiver*” OR “clinic” OR “hospital” OR “ward” OR “individuals” OR “people” OR “adolescents” OR “children” OR “adults”) AND (“health services accessibility” OR “healthcare disparities” OR “access” OR “Health Care Quality, Access, and Evaluation”)	Healthcare
4	1 and 2 and 3	Combining concepts

### Source of evidence selection

A pilot test of our inclusion/exclusion criteria was completed to validate our list of inclusion and exclusion criteria. Two reviewers (Amorim and Wunderlich) independently downloaded the first 100 articles from the PubMed database to the web application, Rayyan (29), and applied the inclusion/exclusion criteria to these articles. Discrepancies were discussed by the first author (Beauchamp) and the second and third authors (Amorim and Wunderlich). Adjustments to the list were not required.

**Figure 1:**
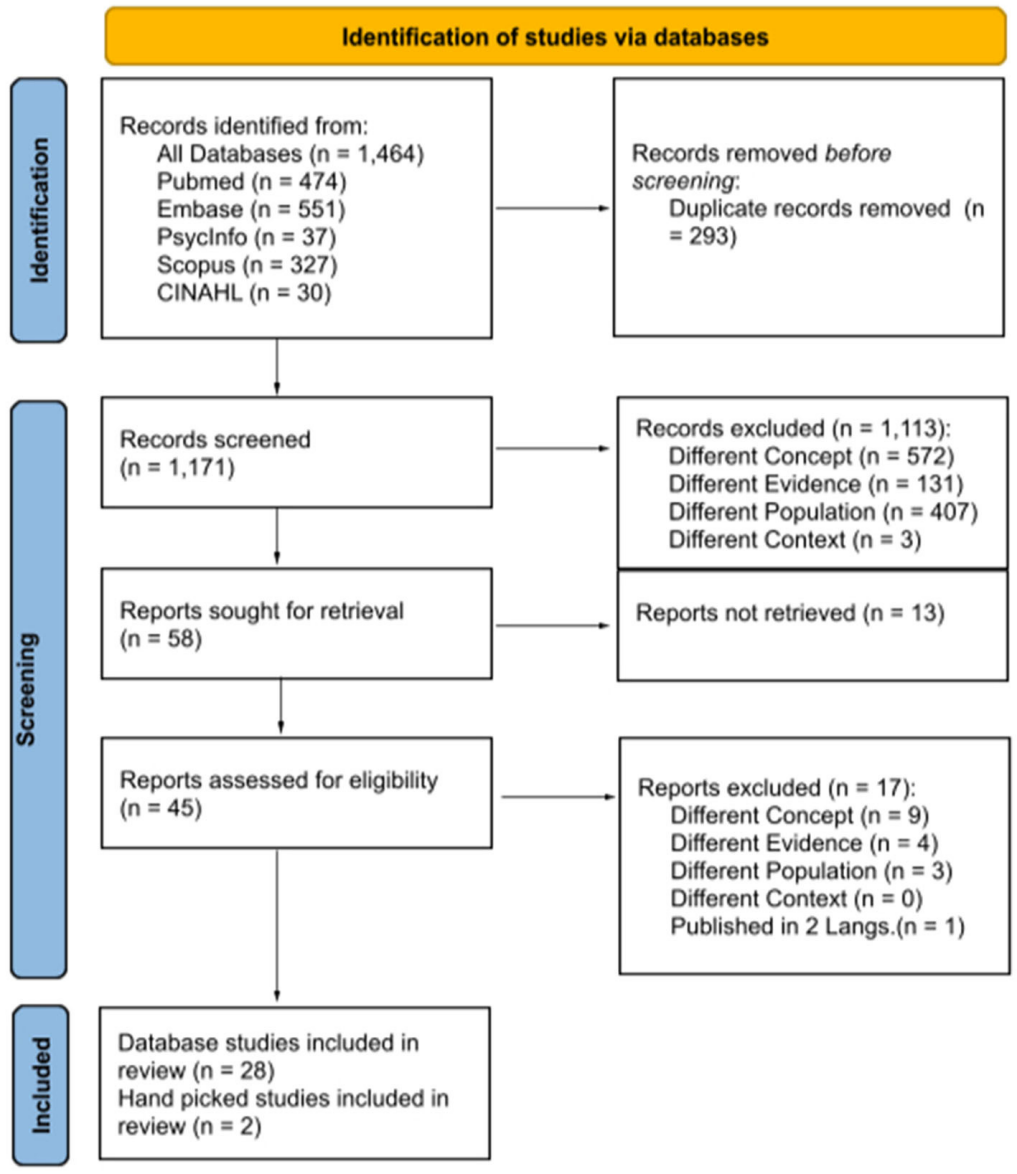
PRISMA 2020 flow diagram of included studies identified via database search and hand pick. From Page et al. (30) https://prisma-statement.org/PRISMAStatement/CitingAndUsingPRISMA.

Full database searches were completed by the two reviewers (Amorim and Wunderlich) on February 21, 2022, and articles were uploaded to Rayyan. After deleting all duplicates, 1,171 articles were retained. Next, titles and abstracts were independently screened against the inclusion/exclusion criteria. For articles that were rejected, reviewers indicated the reason for the rejection (i.e., different population, different concept, different context, or different evidence). In cases where the abstract or title did not permit us to ascertain whether the article met our criteria, the article in question underwent a full text review. The two reviewers' final lists of articles were compared. Disagreements regarding article selection were discussed amongst the first, second and third authors (Beauchamp, Amorim, and Wunderlich). A total of 28 articles met our selection criteria. In addition, two articles that had not been flagged by our search were added based on the first author's knowledge of the literature. The search process and ensuing results are fully reported in Figure 1 of this scoping review using a diagram from the *Preferred Reporting Items for Systematic Reviews and Meta-analyses extension for scoping review* [PRISMA-ScR; (31)].

### Data extraction

Following protocol registration, the two reviewers (Amorim and Wunderlich) independently extracted data from the articles included in our scoping review using data extraction forms. The use of these data extraction forms ensured that data extraction was completed in the same way across the two reviewers. To ensure that our data extraction documents were optimal for this project, the reviewers completed a data extraction pilot: Prior to registering the study protocol, the reviewers independently reviewed the same five articles (one quantitative, three qualitative, and one mixed design) using the data extraction forms, and the data extracted from each article was compared. No changes were required following the data extraction pilot. For the full data extraction process, data were extracted by the second and third authors separately (Amorim and Wunderlich). For each article information about the participants, contexts, and concepts of each article, the methodology used and the findings as they relate to our research question were extracted. Next, the extracted data were compared. Discrepancies were discussed with the first author and resolved. Finally, the extracted data were combined into a single working document to facilitate writing.

## Results

As Table 3 shows, a total of 30 articles were selected: 28 found *via* database search and two through hand search. These include quantitative (*n* = 20), qualitative (*n* = 7), and mixed-model (*n* = 3) studies. Studies were conducted in and across a variety of countries: Australia (*n* = 3), Belgium (*n* = 1), Bulgaria (*n* = 1), Canada (*n* = 6), Denmark (*n* = 1), Egypt (*n* = 1), France (*n* = 1), Germany (*n* = 1), Greece (*n* = 1), Iceland (*n* = 1), India (*n* = 1), Israel (*n* = 1), Malaysia (*n* = 1), Malta (*n* = 1), the Netherlands (*n* = 3), Republic of Ireland (*n* = 1), Singapore (*n* = 1), South Africa (*n* = 2), Sweden (*n* = 3), the United Kingdom (*n* = 5), and the United States of America (*n* = 19) and examined a wide range of issues linked to access and utilization of healthcare services for minority-language speakers with NDDs. These include practitioners' thoughts, feelings and confidence related to serving minority-language speakers with NDDs, their understanding of and adherence to clinical guidelines, and language or geographical concordance with minority-language speakers. Other studies included minority-language parents' and individuals' perceptions on accessing healthcare, factors that influence their ability to access and utilize healthcare, their thoughts, and their beliefs and choices regarding bilingualism as it relates to healthcare access. Participants included: (1) caregivers who were minority-language speakers and who had children diagnosed with an NDD, and (2) service practitioners (mostly doctors, nurses and SLPs) and administrators. Data included in these studies were collected through questionnaires (*n* = 15), interviews (*n* = 7), retrospective record review (*n* = 5), cross-sectional or mixed-mode survey (*n* = 3), focus group (*n* = 2), census data (*n* = 1), and mystery shopper experimental approach (*n* = 1).

**Table 3:** Characteristics of included studies.

**Authors**	**Study objectives**	**Participants**	***N* of** **participants**	**Location**	**Data collection** **methods**	**Domain**		
						**System**	**Provider**	**Family** **experience**
Bergeron et al. (32)	Understand culturally and linguistically diverse parents' perceptions of and practices around their child's language disorder	Parents born outside of Canada who have lived in Canada for less than 20 years	6	Canada	Phenomenological interviews	✓		
Bird et al. (33)	Investigate bilingualism in families with a child with ASD	Parents in a bilingual family who have one or more children with ASD	49	Canada, USA, Greece, France, Egypt, and Singapore	Questionnaire	✓	✓	
Caesar et al. (34)	Investigate the frequency SLPs used recommended practices when assessing bilingual students	Public school clinicians in Michigan	130	USA	Questionnaire	✓	✓	
de Valenzuela et al. (35)	Examine issues related to the inclusion and exclusion of students with developmental disabilities in and from bilingual opportunities	Policy makers, professionals, and practitioners in special needs and/or language education	79	Canada, USA, UK, the Netherlands	Semi-structured, one-on-one interviews	✓		
Fong et al. (36)	Document the barriers and facilitators Korean immigrant families encounter when accessing autism-related services	Korean parents of children with ASD	20	Canada	Individual semi-structured interview	✓	✓	✓
Hammer et al. (37)	Determine the level of training and confidence of SLPs in serving Spanish–English bilingual children	SLPs	213	USA	Questionnaire	✓		
Jimenez et al. (38)	Determine the national average wait time for developmental pediatric evaluations, and to understand differences in access	Developmental, neurodevelopmental, developmental-behavioral, or developmental disability clinics	90	USA	Mystery shopper study *via* phone calls	✓		
Jordaan (39)	Establish caseload characteristics, language profiles and proficiencies, and practices of SLPs regarding bilingual clients	SLPs providing intervention to bilingual children	99	Israel, Malta, Belgium, India, Canada, USA, UK, Sweden, Malaysia, Bulgaria, Denmark, Iceland, and South Africa	Questionnaire	✓	✓	
Kritikos (40)	Determine SLPs' beliefs about language assessment of bilingual/bicultural individuals	SLPs	811	USA	Questionnaire	✓	✓	
Kuhn et al. (41)	Examine child and family factors that predict receiving a diagnostic evaluation and a confirmed ASD diagnosis	Medical Records of racial/ethnic minority children who screened positive for ASD	309	USA	Retrospective medical record review	✓		
Marinova-Todd et al. (42)	Gather information from professionals about their practices and opinions pertaining to the provision of bilingual supports to students with developmental disabilities	SLPs, teachers, language specialists, early childhood educator, administrators, and other professionals	361	Canada, USA, UK, and the Netherlands	Questionnaire	✓	✓	
Mcleod and Baker (43)	Describe practices regarding assessment and service delivery for children with speech sound disorders	SLPs	231	Australia	Questionnaire	✓	✓	
Mennen and Stansfield (44)	Identify the level to which SLP services meet recommendations; to examine caseloads; and to determine whether services are in place to meet the needs of those bilingual clients	SLPs and their managers	21	UK	Questionnaire and interviews	✓		
Mulgrew et al. (45)	Investigate the perceptions and practices of SLPs in the assessment of bilingual English–Irish-speaking children	Community-based practicing pediatric SLPs	181	Republic of Ireland and UK	Online cross-sectional survey	✓	✓	
Nayeb et al. (46)	Investigate nurses' perceptions of language screening and their practice for bilingual children	Nurses who perform language screening of bilingual children.	863	Sweden	Online questionnaire	✓	✓	
Pascoe et al. (47)	Investigate assessments and interventions used by SLPs in the Western Cape when working with children with speech difficulties	SLPs working with children	28	South Africa	Questionnaire	✓	✓	
Rethfeldt (48)	Examine current provision of speech-language intervention services for multilingual children	SLP practices	28 SLP practices	Germany	Cross-sectional survey	✓	✓	✓
Rodrigues et al. (49)	Compare the receipt of developmental surveillance and screening among children	Medical records of children who attended wellness child visits	450	USA	Retrospective medical record review	✓	✓	
St Amant et al. (50)	Examine the influence of current ethnic and acculturation differences, with an emphasis on parental primary language, on child involvement in ASD-specific services	Medical records of children receiving services with a confirmed individualized education plan	152	USA	Retrospective medical record review	✓		
Vanegas (51)	Examine child, maternal, and family-level factors on the age of first autism spectrum disorder diagnosis among a diverse, clinical sample	Medical records of children with ASD	221	USA	Retrospective medical record review			
Verdon et al. (52)	Make a geographical comparison between multilingual children and multilingual SLP services in Australia	SLPs and children aged 4–5 years old	2849 SLPs; 4386 children	Australia	Questionnaire and census data	✓		
Wiefferink et al. (53)	Understand factors that may influence early identification by providing a detailed description of caseload characteristics	Medical records of children with language difficulties	9932	Netherlands	Retrospective medical record review	✓	✓	
Williams and McLeod (54)	Examine Australian SLPs' perspectives and experiences of multilingualism, including their assessment and intervention practices	SLPs working with multilingual children in Australia in 2010	128	Australia	Questionnaire	✓	✓	
Yu (20)	Explore the influences for and the effects of the language choices made by the mothers in relationship to their children with ASD	Parents who spoke Mandarin Chinese and had a child with ASD	10	USA	Phenomenological interviews	✓	✓	
Yu and Hsia (55)	Examine the constraints and affordances of heritage language maintenance efforts in three families of children on the autism spectrum	Parents who have children with ASD and identify Chinese as a heritage language	3	USA	Interview	✓	✓	
Zuckerman et al. (56)	Develop and test a brief, English/Spanish bilingual parent-reported scale of perceived community ASD stigma	Parents of children with ASD aged 2 to 10 years old	380	USA	Questionnaire	✓		✓
Zuckerman et al. (57)	Compare barriers to ASD diagnosis and current ASD-related service use among families with English proficiency or limited English proficiency	Parents of children with ASD.	352	USA	Mixed-mode survey	✓		✓
Zuckerman et al. (58)	To assess ASD and developmental screening practices, attitudes toward ASD identification in Latino children, and barriers to ASD identification for Latino children	California pediatricians	267	USA	Questionnaire	✓	✓	
Zuckerman et al. (59)	To assess barriers to ASD diagnosis in the Latino community	Parents of children with ASD	33	USA	Focus group	✓		✓
Zuckerman et al. (60)	To qualitatively assess the potential barriers of ASD in the Latino community	Latino parents of typically developing children	30	USA	Focus group or individual semi-structured interview	✓		

### Data analysis and presentation

Data included in the analysis reflects our research question. Based on the data extraction, we identified barriers to healthcare access and utilization for individuals with an NDD and their families who are minority-language speakers. For each barrier we also identified by which participants it was reported (e.g., individuals, clinicians, parents), and the context (e.g., psychologists in schools).

The reader should note that throughout, we use the term “minority-language speakers” to refer to (a) individuals who speak only a minority language and who have received little to no exposure to the majority language and (b) to bilinguals since for them, one language is generally a minority language. We also use the term “bilingual” to refer to children who are exposed to two (or more) languages, even if they are not fluent in these languages or are in the process of acquiring them.

### Barriers

As Table 4 shows, our review revealed several barriers to accessing and utilizing healthcare services. We classified them into three main domains: Systems, Practitioners, and Family Experience. In the following, we discuss barriers identified within each of the three domains and note the number of articles in which the target barrier was identified.

**Table 4:** Thematic barriers by domain found in included studies.

**Barriers**	**Domain**			**Citations**
	**System**	**Provider**	**Family** **experience**	
1. Disconnect between the needs of users and services offered	✓			(20, 32–35, 38, 40, 42, 48, 52, 55)
2. Quality of treatment	✓			(34, 37, 40, 41, 46, 48–50, 53, 54, 56–58)
3. Lack of training for healthcare professionals	✓			(20, 33, 34, 37, 39, 40, 44, 46, 47, 54, 55)
4. Difficulties accessing interpreters	✓			(34, 37, 39–41, 43, 45, 48, 58–60)
5. Lack of available information in minority languages	✓			(32, 36, 48, 57, 58, 60)
6. Personal characteristics of healthcare practitioners		✓		(40, 58)
7. Practitioners' often erroneous beliefs regarding language development		✓		(20, 33, 39, 40, 46–48, 55)
8. Practitioner's lack of using evidence-based practices		✓		(34, 39, 42, 43, 45, 47, 48, 53, 54, 58)
9. Challenges in offering family-centered services		✓		(36, 39, 54)
10. Lack of resources		✓		(34, 39, 40, 45, 46, 49)
11. Feelings of distrust toward language discordant healthcare providers			✓	(36, 57, 59)
12. Feelings of stigma			✓	(48, 56)

#### Systems

This domain refers to policies, procedures, or practices that tend to have a negative impact on access or utilization of healthcare services (36). Within this domain, five main themes emerged: (1) disconnect between the needs of users and services offered, (2) treatment quality, (3) lack or training for healthcare professionals, (4) challenges with interpretive services, and (5) lack of quality information in the minority language.

The first barrier within this domain is the *disconnect between the needs of users and services offered* (11 studies). This barrier is evidenced first and foremost by the lack of services offered in languages other than the majority language, and by the lack of interventionists who speak a language other than the majority language (20, 33, 34, 40, 52). Indeed, while caregivers report wanting services in their minority-language, they also report challenges in finding intervention support in that language (20, 32, 33, 55). What is striking is that the need for services in minority languages may not be recognized by those who work within the healthcare system. For example, in a study of SLPs in Germany, only 40% of practitioners considered that service options for minority-language speakers were inadequate (48), which seem to conflict with the challenges in finding services in the minority-language that parents experience. Sadly, challenges in finding services in the minority language have led some caregivers to make the difficult choice to only speak the majority language with their child with an NDD, believing that their child would otherwise “lose out” on receiving intervention (20). This barrier is also linked to findings that access to services for minority-language speakers with NDDs are seldom prioritized (35, 42) and that access to a language other than the majority language is viewed as being separate from, rather than integrated into, special needs services (35). What is particularly concerning is that frequently, programmes that offer services for children with NDDs, including those that are publicly funded, do not make accommodations for minority-language speakers despite regulations and guidelines that call for such accommodations [such as the Title VI of the Civil Rights Act and National Standard for Culturally and Linguistically Appropriate Services in the USA; (38)]. Such barriers lead to children with NDDs from minority-language families experiencing important challenges when trying to access and use healthcare.

Even when minority-language speakers with NDDs are able to access healthcare services (be it in the majority or the minority language), they often experience barriers with regards to the *quality of the treatment* that they receive (13 studies). Indeed, children from minority-language households are less likely than their majority-language peers to receive a developmental screener (41, 49, 58), or a referral for a language or developmental assessment from their pediatrician (46, 48, 49, 53). Consequently, children from minority-language households tend to receive an NDD diagnosis later than their majority-language peers [(48), although see (47) for contradicting findings]. Additionally, minority-language children may be offered fewer hours of intervention services (50, 57) when compared to majority-language speakers, and their intervention plans tend to include fewer social-skills goals (50, 56–58). Of note, these findings were maintained even after controlling for various demographic variables such as socio-economic status. Interestingly, language-based disparities may differ based on the social standing of the speaker's language. For example, in a study completed in Denver, Colorado, USA, Rodrigues and colleagues found that children who spoke neither English nor Spanish were less likely to receive developmental screening than Spanish-speaking or English-speaking children [while there was no difference between these two groups; (49)]. These disparities likely reflect differences in the social standing of the Spanish language in the study's geographic context compared to the standing of other minority languages. Indeed, according to the Census Bureau, Hispanics, and Latinos make-up ~29% of the population in Denver (61), and of those, 57% report speaking Spanish at home (62). Because of Spanish's relatively high representation in the community, practitioners were able to access more materials and tools in Spanish compared to other minority languages, which limited the disparities between Spanish and English-speaking children (49). Taken together, minority-language speakers with NDDs face important challenges in access and utilization of services. However, other factors such a lack of professionals trained to work with minority-language children with NDDs (34, 37, 40, 46, 54) also create important barriers for these children.

While studies may suggest that it is preferable for minority-language speakers to work with interventionists who speak their minority language (37, 44), it is not always possible for them to do so. However, even when practitioners do not speak their client's minority language, they can work with them and support the development of this minority-language. Nonetheless, many interventionists express feeling unprepared to work with minority-language speakers with NDDs and their families (34, 37, 46, 54). This feeling is likely the result of the next barrier, the *lack of training for healthcare professionals to better support minority-language speakers* (11 studies). Practitioners frequently report receiving no instruction regarding the administration of language screening tools and the interpretation of ensuing results when assessing bilingual children, while the vast majority also report receiving such training to assess majority-language monolingual children (46). Although these findings may not be surprising when thinking about healthcare practitioners such as pediatricians and nurses, it is surprising to find similar trends for SLPs. Indeed, SLPs frequently report not receiving adequate training to work with minority-language families during their graduate studies (34, 37, 54) and beyond their university training (37, 54). For example, Pascoe and colleagues found that SLPs frequently rate their level of confidence to work with children from bilingual households as being rather low, even when they have had many years of experience (47). What is particularly troubling is that SLPs report a lack of mandatory training, even in contexts where their caseloads include several minority-language children (44). This lack of training for healthcare practitioners has led to challenges in correctly identifying language disorders in children from minority-language families (39, 40, 47). It has also resulted in caregivers receiving inconsistent or erroneous advice regarding whether they should raise their child in a bilingual context (20, 33, 39, 55). It is therefore not surprising that many families have made the heartbreaking choice to avoid speaking their minority-language with their child with an NDD which has important repercussions for language, communication, and psychosocial development [see (18) and (19) for discussion].

Beyond access to training, *difficulties in accessing interpreters* (11 studies) is also frequently cited as a barrier to accessing healthcare services (34, 39–41, 43, 48, 58–60). Caregivers report that interpreters are often unavailable and when they are available, it may only be by telephone, which caregivers regard as inappropriate and inadequate given the sensitivity of the subjects discussed with the practitioner (41, 60). The lack of interpreters hinders effective communication between caregivers and practitioners. Most notably, caregivers report that the lack of access to interpreters leads to challenges in scheduling appointments and in navigating the healthcare system (59). Caregivers also report that interpreters are often poorly trained (59, 60), which creates additional barriers to their ability to express their concerns regarding their child's development. Even when interpreters are professionally trained, they seldom have received training to support healthcare workers in their work [for example supporting SLPs during speech and language assessments (39, 45)]. Finally, practitioners who infrequently work with interpreters (trained or otherwise) may find it challenging to do so, particularly when they've not received training as to how to work effectively with interpreters (40). Unfortunately, findings suggest that few practitioners receive this type of training (37, 40).

In addition to the lack of interpreters, caregivers who are minority-language speakers also report a *lack of available information in minority languages* (six articles). Parents often have difficulty accessing printed information in their minority language about their child's disorder (60) and guidance or information regarding the various services and resources available to their family (36). And, even when materials are available, their quality is often poor (60). Moreover, because materials offered in the majority language often include medical jargon, they are difficult for minority-language caregivers to understand [particularly for those caregivers who have limited levels of proficiency in the majority language; (60)]. Consequently, caregivers who have limited proficiency in the majority language are less likely to seek out the information about their child's disorder and have more challenges understanding the information obtained (36) which leads them to have lower levels of knowledge about NDDs (32, 48, 57, 58, 60) than majority-language caregivers. This lack of information regarding child development and NDDs may lead to further delays in children with NDDs being identified (60).

Overall, the barriers identified in this domain reflect that sad truth that the needs of minority-language speakers with NDDs and their families are less likely to be met compared to those of majority-language peers and their families (56, 57). These unmet needs include the lack of intervention services or other care services, and the lack of adequate information (56, 57, 59). Unfortunately, the barriers within the System's domain are not the only barriers that minority-language families face in accessing and utilizing healthcare. They also face barriers from practitioners themselves.

#### Practitioners

The second domain refers to barriers related to the characteristics and beliefs of practitioners. Such barriers emerged in 17 studies and include: (1) characteristics of the practitioners, (2) practitioners' (erroneous) beliefs regarding language development in minority-language children with NDDs, (3) the lack of evidenced-based practices (4) challenges in offering family-centered services and (5) the lack of resources required to work with minority-language children with NDDs.

The first barrier within the Practitioners domain is defined as the *characteristics of the practitioners* (two articles). One such characteristic is the lack of linguistic diversity. In their study, Zuckerman and colleagues found that 56% of primary care practitioners who worked with minority-language speakers listed language discordance (i.e., practitioners speaking a language that is different from their patient's language) as a barrier to working effectively with their patients (58). A second characteristic identified as a barrier is a practitioner's caseload and the linguistic background of the other practitioners within their practice, which has an influence on their ability to work effectively with minority-language speakers. For example, pediatricians who have few minority-language patients (<25% of their caseload) are less likely to feel confident in identifying signs of autism in minority-language children (58). And those whose practice does not include colleagues who were minority-language speakers were less likely to offer developmental or autism screenings to children from minority-language backgrounds (58). Kritikos and colleagues also found that SLPs who identified as monolinguals reported feeling less proficient when working with an interpreter than bilingual SLPs (40). Thus, a practitioner's personal characteristics can influence the services rendered.

A second barrier to emerge was *practitioners' often erroneous beliefs regarding language development in minority-language in children with NDDs* (eight articles). Findings indicate that healthcare practitioners are often weary of recommending that children with NDDs be exposed to two languages, even when these children are from minority-language households (20, 33, 39, 55). This weariness seems to stem from the belief that in children with NDDs, particularly those that affect language and cognition, exposure to two languages will cause additional delay in language development (46). Thus, practitioners often recommend that families refrain from exposing children with NDDs to a second language. Given language constraints related to the language of schooling and of intervention, minority-language families are therefore generally told to avoid using their minority/heritage-language with their child in favor of the majority-language (20, 33). As previously discussed, this practice is not evidence-based and may even be detrimental to children's development [(19), also see (18) for discussion]. In addition, practitioners are also less likely to refer children from bilingual households for SLP services either because they are cautious of over-referring and/or due to their lack of knowledge regarding language development in minority-language children (40, 46, 47). Such practices could have important consequences for children's outcomes and families' well-being. The lack of understanding regarding language development in minority-language (either who are single or dual/multi-language learners) children also leads to challenges in assessing their language abilities. When conducting a language assessment with minority-language speakers, it is important to differentiate between children who have not yet completely acquired the majority language because they have not been sufficiently exposed to this language, from children who have a true language disorder (39, 40, 47). When practitioners do not have a solid understanding of bilingual development in children, they are more likely to identify a language disorder in children who are simply in the process of acquiring the language in which they were tested. For example, Rethfeldt (48) found that 57% of minority-language children who were diagnosed as having a DLD by a pediatrician were judged by SLPs as simply having a delay in the acquisition of the majority language rather than a true DLD, indicating overdiagnosis of DLDs on the part of pediatricians. In contrast, likely because of minority-language children's incomplete acquisition of the majority-language, practitioners may be less prone to identify the early signs of NDDs in children these children and therefore may not refer them to services in a timely manner (40, 46).

While false beliefs and a lack of understanding of language development in minority-language speakers likely stems from a lack of training, even when training is available practitioners do not consistently *use evidence-based practices*; another barrier emerging in 10 articles. For various reasons, too many practitioners are not using evidence to inform their practice when working with minority-language children with NDDs. For example, Pascoe et al. (47) report that even when best-practice guidelines are available, practitioners do not consistently use them to assess bilingual children. Other studies show that while practitioners may believe that they are using best practices, in reality, the methods they are using do not follow best-practices (42, 47, 54). Crucially, assessing minority-language children (who are either monolinguals or bilinguals) requires different strategies to those used when assessing majority-language children, as well as cultural competency (48) [see (63) for discussion on bilingual assessments]. Yet, many practitioners report relying solely on standardized measures in the majority language when assessing these children (34, 43, 45, 48, 54, 58) and many report not using an interpreter when assessing minority-language children (34, 39, 45, 48). While departures from best practices may be due to a lack of training, they may also be due to external factors like policies requiring standardized scores or time constraints (45). Whatever the underlying cause, such practices can lead to delays in diagnosis or to misdiagnoses (53).

*The challenge of offering family-centered services* is another barrier in the Provider domain (found in three studies). Indeed, despite recognition of its importance for minority-language speakers with NDDs (36, 54) practitioners can face difficulties adapting to the cultural expectations of each of their clients, being sensitive to diverging attitudes toward disabilities, and in reconciling their own beliefs with those of families [see (54) for an entire list of these challenges]. While these challenges may not be specific to minority-language services, they are likely common when working with minority-language families (36, 54). Practitioners' recommendation against bilingualism (39), despite families wanting to maintain their minority language (39) is also a challenge to family-centered practice, in addition to contravening best-practices. Consequently, community organizations are often called upon to address many cultural and language barriers that are typically ignored by traditional healthcare practitioners, by offering information in the minority language, emotional support, and guidance (36). The need for such services highlights the need for the integration of family-centered approaches within the healthcare system.

The final barrier linked to practitioners is the *lack of resources* (six studies) required to offer high quality services to minority-language speakers with NDDs and their families. This includes financial resources, physical resources (such as assessment tools), and time (34, 39). For example, studies note the prohibitive costs linked to hiring interpreters and practitioners who are trained to offer services to minority-language speakers (34, 39). Other studies note the lack of assessment and intervention tools in minority-languages (40, 45, 46, 49). Finally, while best-practices include working with trained interpreters (64), this practice can be more time consuming than working directly with a caregiver who speaks the majority language (49), as are bilingual language assessments (45). In certain contexts, practitioners may not be allowed this extra time as it may come at the expense of seeing other patients (34, 39).

#### Family experience

The last of the three domains refers to barriers linked directly to the family's experience accessing and using healthcare services. Two barriers emerged in the literature: (1) distrust toward healthcare practitioners who do not speak their minority language and (2) stigma.

Findings indicate that minority-language families often experience *feelings of distrust toward healthcare practitioners who do not speak their minority language* (three studies). Such distrust is the result of the inappropriate ways in which practitioners interact with caregivers from minority-language backgrounds. First, caregivers report encountering practitioners who are unable to provide information in a way that is culturally and linguistically appropriate (36, 57, 59). Caregivers also report experiencing discrimination from practitioners (36), which likely arises from a lack of cultural sensitivity. Finally, caregivers' distrust of practitioners who do not speak their minority-language may be a result of the frustrations that they experience during the diagnostic process, which in turn leads to doubts as to whether the provider was acting in families' best interests (59). Unfortunately, these feelings may lead to parents delaying acting on the recommendations of practitioners which they deemed less trustworthy (59).

Minority-language speakers with NDDs and their families also experience *stigma* when utilizing healthcare services (in two articles). Indeed, families with limited proficiency in the majority language have reported significantly higher rates of stigmatization compared to individuals who were visible minorities but majority-language speakers (56). The experience of stigma persisted after controlling for socio-economic status and was associated with unmet needs in treatment services (56). Unfortunately, the experience of stigma appears rampant in some communities; 77% of clinicians described their minority-language clients as being at a high risk of stigmatization (48).

## Discussion

This scoping review examined barriers to the access and utilization of healthcare services for individuals with NDDs who are minority-language speakers and their families. Findings from the studies reviewed show that individuals in this minority group face numerous barriers in accessing and utilizing healthcare services. Overall, we identified 12 different barriers linked to Systems, Practitioners, and Family Experience, summarized in Table 4.

For the most part, our findings examining barriers faced by minority-language speakers with NDDs converge with those found in studies examining minority-language speakers' access and utilization of healthcare more broadly. Common to both groups is the language discordance between practitioners and patients, which leads to communication breakdowns and increased patient stress (6, 58). Moreover, limited availability of services in minority-languages also leads to lower quality of care and longer wait times (8, 9, 20, 33, 34, 40, 52). Minority-language speakers also consistently report discrimination and negative attitudes on the part of healthcare practitioners (7, 10, 36) and challenges linked to access to qualified interpreters (7, 34, 36). However, our findings highlight that barriers facing minority-language speakers, are also amplified for those with NDDs and their families. Key barriers for this population include practitioners' erroneous beliefs about language development in minority-language children with NDDs, and the lack of training for healthcare professionals to better assess and support the speech and language of individuals in this subgroup.

Evidence shows that erroneous beliefs regarding bilingual language development in children with NDDs leads to several negative consequences for minority-language speakers. First, practitioners often recommend against exposure of these children to their native languages or to the languages used by their family (20, 33, 39, 55). Second, practitioner may delay referrals to developmental or language assessments, or may not correctly identify a child as having a language disorder because they assume in children's language development will be due bilingualism (46, 48, 49, 53). Consequently, minority-language speakers are at increased risk for both delays in receiving an NDD diagnosis and accessing appropriate intervention. Conversely, minority-language speakers are also at risk of being misdiagnosed as having speech and language disorders when they are in fact in the process of acquiring the majority-language.

It is striking that erroneous beliefs about bilingual development persist despite a wealth of evidence highlighting that bilingualism does not cause language delays, that minority-language speakers with NDDs can become bilinguals and that for all children, but that these children's abilities in each of their languages will be influenced by several factors such as the amount of language exposure that they receive in each language (25, 65). Furthermore, in addition to interfering with quality of care, recommendations against these children being exposed to their native language also reduce resilience in subgroups like immigrants and refugees by leading to further loss of kinship and community tradition (66). The persistence of these erroneous practices, despite the seriousness of their consequences likely reflects persistence in biases and/or a lack of training opportunities regarding language development in minority-language children. The fact that many studies in this scoping review focused on autism specifically may suggest that some of the biases faced by minority-language speakers may be greater for individuals with autism from minority families than for other families with NDDs, although the extent to which this is be true is beyond the scope of this review.

Although our review focused on identifying barriers to access and utilization of healthcare services faced by minority language speakers, key solutions that can mitigate these barriers and level the playing field for minority language speakers are equally evident. Next, we present five key areas emerging from our review, where improvements are needed and can be feasibly implemented. Specifically: the development and uptake of policies and guidelines, practitioner training, referral pathways for specialized services, access to appropriate tools and resources, and partnership with caregivers.

First, there is a need for healthcare systems to develop and implement *policies and guidelines that support minority-language speakers*. One proposed policy is to increase the number of bilingual practitioners within the healthcare system by ensuring the inclusion of culturally and linguistically diverse practitioners (34, 41, 57). Where possible, increasing language concordance between users and practitioners may decrease feelings of mistrust toward practitioners who do not speak their minority language (57). Increasing concordance can also improve access to information and resources for minority-language speakers (36, 57) and therefore increase their knowledge about their child's disorder.

Overall, there is also a need for policies that ensure the adoption of evidence and best-practices through the development of evidence-based clinical guidelines where they do not exist, and measures to ensure that practitioners are following existing guidelines (34, 37, 38, 44, 58). New or existing guidelines can also benefit from adopting standards in how bilingualism is defined and how equity in service delivery is achieved for minority-language speakers (45). It is also critical that policies do not contradict best-practices. For example, practitioners should not be required to use specific standardized tests or to provide standardized scores to enable families to access services, financial supports, or reimbursement for services when the use of such measures is counter indicated. Rather qualified practitioners should be authorized to provide other types of information to describe children's language abilities (45).

Second, practitioners who work with minority-language speakers must *receive appropriate mandatory training* (34, 37, 40, 44–46, 48, 53, 54, 60). Given the consequences of practitioners' lack of training discussed above (e.g., delays in services, misdiagnosis), training is key to improving care for minority-language speakers with NDDs and their families. Indeed, findings show that many practitioners feel insufficiently trained to work with minority-language speakers (34, 46, 54) and less competent when they work with this population (37, 46, 47). Other findings indicate a lack of awareness on the part of practitioners regarding their need for training in this area (37). Therefore, mandatory training (as opposed to voluntary training) is likely the best way to counter the Dunning–Kruger effect [i.e., the effect which suggests that individuals with low levels of knowledge overestimate their abilities (67)].

Universities have a key role in preparing future healthcare professionals to work with minority-language speakers, including those with NDDs. To address the shortage of practitioners who speak a minority language, universities can ensure the inclusion of minority-language students in their training programmes (39, 54) and facilitate language learning opportunities for all their students (34). University programs can also enhance their core curriculum to include up to date evidence on bilingual language development in typically developing children and in children with NDDs, as well as cultural competency, among other areas of knowledge and skills relevant for practice with minority-language speakers.

Employers should also ensure that their employees are adequately trained to work with minority-language speakers (with and without NDDs). Innovative approaches to training can improve practitioners' awareness of the challenges of working with minority-language speakers with NDDs and mitigate biases especially during assessments and when working with interpreters (34, 36, 39, 52, 54). Training should also help practitioners develop cultural competency, especially since it is not always possible for practitioners to be fluent in all of the languages they will encounter (52). Finally, training should be appropriate to the practitioners' responsibilities. Thus, some practitioners, like SLPs, may require more specialized training to work with minority-language speakers (for example, having a strong understanding bilingual language develop and of best-practices when assessing minority-language speakers' language abilities).

In sum, training is key to increasing practitioners' understanding of bilingual language development, diminish false-beliefs and ensuring that practitioners are adequately trained given their responsibilities (34, 37, 45, 46, 52). It also decreases the Dunning–Kruger effect, and thus it increases the likelihood that children from minority-language families are adequately referred to practitioners who specialize in assessing and working with minority-language children with NDDs. Training also may go a long way to help diminish stigma by enhancing trust and family-centered care (36, 41, 48, 56, 57, 59).

The third area of improvement is ensuring that practitioner *refer minority-language speakers to specialized practitioner when there is any doubt regarding children's language or general development*. For example, minority-language children with (or suspected of) speech or language disorders, should be referred to an SLP who specializes in working with this population. By referring children to the appropriate services and to professionals who are experienced and knowledgeable in working with minority-language children with NDDs, these children are less likely to be misdiagnosed and are more likely to receive the types of services that they genuinely require (46, 48). It also ensures that minority-language children do not experience additional delays in accessing assessment and intervention services, and that intervention goals, particularly those in the areas of social skills and language, reflect their needs and are adequately targeted. Thus, to facilitate appropriate referral practices, practitioners are strongly encouraged to work collaboratively in multidisciplinary frameworks (48).

The fourth area of improvement is to ensure that practitioners have access to the necessary *tools and resources necessary to enable them to follow best practices learned during training and outlined in guidelines*. This includes access to trained interpreters who can play a variety of roles including facilitating access to services and other community resources, and helping families to communicate with practitioners (36, 45). However, it is important that interpreters be adequately trained [i.e., have knowledge in working with medical professionals, and have knowledge in NDDs (34, 36)] in order to effectively support both families and healthcare professionals. Beyond the access to trained interpreters, there is a need for screening and assessment tools in multiple languages (45, 47, 49). Recent studies across the globe have highlighted the importance of developing standardized tools to support accurate identification of language disorders and other NDDs, while simultaneously favoring culturally sensitive and contextually appropriate use of existing tools, even when those were not normed or developed for a specific language group (68). Such practices, relevant for all NDDs, include working with interpreters during assessments, obtaining information about the client's culture and language exposure, collecting information about the child's productions in their first language, and the use of dynamic testing (39, 42, 45–48, 54). Additionally, while there is a significant amount of available information regarding language development for some languages like English, there are fewer published norms for other languages (47). Therefore, researchers are called upon to develop language norms for these languages and to ensure that these norms are readily available in several languages (rather than only in the target language). Use of such practices and an increase in resource's is likely to reduce the risks of over- or under-diagnosing a language disorder in minority-language children. Employers must also ensure that practitioners are given the time to implement best-practices when working with minority-language individuals with NDD.

Finally, caregivers are a wonderful resource who need to be viewed by healthcare practitioners as *partners*. One important way to increase the partnership between caregivers and practitioners is for healthcare services to engage in stakeholder engagement activities to better identify the barriers in their context and ways to mediate these barriers. Stakeholders should include individuals with NDDs who are minority-language speakers and their caregivers, as well as practitioners and other frontline workers, and administrators. By engaging the different stakeholders, healthcare systems will better understand the situation in their individual context and find solutions that best suit the needs of their stakeholders. Effectively working with caregivers also includes supporting them to work with their children (54). This can include offering caregiver training and using parents as partners in the intervention process, by directly involving them in their child's intervention (54). These strategies increase parents' empowerment through increased understanding of their child's condition and improved access to services and resources. They also help minority-language caregivers implement intervention strategies at home in their minority language.

Taken together, the areas of improvement we proposed would enhance person- and family-centered care, and lead to improvements in the quality and efficiency of healthcare services. More importantly, implementing such solutions will begin to address the clear inequity facing minority language speakers. Future research is also needed to examine the efficacy of the proposed solutions in mitigating barriers in a various regions of the world and in different healthcare contexts. Differences in populations and systems might favor some solutions over others. For example, what are key differences in healthcare services that minority-language children with NDDs receive based on whether their minority-language is an official or unofficial language? New and unique solutions may be required for minority-language speakers who also face significant stigma and marginalization and/or those who are survivors of trauma. Furthermore, our review has highlighted a striking lack of studies with first person perspectives of people with NDDs themselves. Future research can build on recent advocacy and improved methods for capturing these perspectives (69, 70) in order to build a more accurate picture of the lived experience of minority language speakers in healthcare.

## Author contributions

MB developed the research question, protocol, wrote the protocol for registration, and wrote the manuscript. MB, KA, and SW developed search terms, inclusion/exclusion criteria, data extraction forms and responsible for major manuscript revisions. KA and SW screened articles and completed data extraction. MB, KA, SW, JL, JS, and ME provided feedback for the protocol and revisions for the protocol and manuscript. All authors contributed to the article and approved the submitted version.

## Funding

This project is funded by the following grant: Health Canada, and supported by Dialogue McGill at McGill University, under the Action Plan for Official Languages— 2018–2023: Investing in Our Future, a grant from the Fonds de Research du Québec en Sciences (#34734), and by the *Toronto Dominion Bank Post-Doctoral Fellowship in Child Health Research Excellence*.

## Conflict of interest

The authors declare that the research was conducted in the absence of any commercial or financial relationships that could be construed as a potential conflict of interest. The reviewer FG declared a shared parent affiliation with the authors MB, KA, SW, JS, and ME to the handling editor at the time of review.

## Publisher's note

All claims expressed in this article are solely those of the authors and do not necessarily represent those of their affiliated organizations, or those of the publisher, the editors and the reviewers. Any product that may be evaluated in this article, or claim that may be made by its manufacturer, is not guaranteed or endorsed by the publisher.
